# Macrophage metabolism in nonalcoholic fatty liver disease

**DOI:** 10.3389/fimmu.2023.1257596

**Published:** 2023-10-04

**Authors:** Wenhui Zhang, Ren Lang

**Affiliations:** Department of Hepatobiliary Surgery, Beijing Chao-Yang Hospital Affiliated to Capital Medical University, Beijing, China

**Keywords:** nonalcoholic fatty liver disease, macrophage, immunometabolism, polarization, activation, therapeutic target

## Abstract

Nonalcoholic fatty liver disease (NAFLD) and its inflammatory and often progressive subtype nonalcoholic steatohepatitis (NASH), have emerged as significant contributors to hepatic morbidity worldwide. The pathophysiology of NAFLD/NASH is multifaceted, variable, and remains incompletely understood. The pivotal role of liver-resident and recruited macrophages in the pathogenesis of NAFLD and NASH is widely acknowledged as a crucial factor in innate immunity. The remarkable plasticity of macrophages enables them to assume diverse activation and polarization states, dictated by their immunometabolism microenvironment and functional requirements. Recent studies in the field of immunometabolism have elucidated that alterations in the metabolic profile of macrophages can profoundly influence their activation state and functionality, thereby influencing various pathological processes. This review primarily focuses on elucidating the polarization and activation states of macrophages, highlighting the correlation between their metabolic characteristics and the transition from pro-inflammatory to anti-inflammatory phenotypes. Additionally, we explore the potential of targeting macrophage metabolism as a promising therapeutic approach for the management of NAFLD/NASH.

## 1 Introduction

Nonalcoholic fatty liver disease (NAFLD), a prominent global public health concern, is projected to surpass all other indications for liver transplantation in the United States by 2020 (1, 2) . A subset of individuals with NAFLD progresses to a more inflammatory condition known as nonalcoholic steatohepatitis (NASH), which can further advance to severe liver fibrosis, cirrhosis, or hepatocellular carcinoma (HCC). Extensive research has been dedicated to understanding the pathogenesis of NAFLD and NASH, highlighting the significant involvement of innate immunity (3–6). Within this context, macrophages play a pivotal role in the innate immune response and are indispensable for the development of NAFLD and NASH (7).

Immunometabolism, currently a burgeoning field of research, focuses on investigating the metabolic processes of immune cells and exploring the effects of modifying their metabolic phenotype on their functionality (8–10). The functional behavior of immune cells is intricately regulated by the microenvironment, which, in turn, exerts a profound influence on their metabolism (11). Cytokines, growth factors, and various environmental signals play a crucial role in modulating the metabolism of immune cells. Emerging evidence suggests that macrophages undergo metabolic reprogramming in specific microenvironments, particularly in inflammatory conditions such as NAFLD/NASH, to meet their specific requirements and execute effector functions, such as phagocytosis and cytokine production (4, 12, 13). Exerting control over the metabolic activity of macrophages holds immense promise in their engagement in inflammatory conditions. Therefore, comprehending the metabolic processes and regulatory common mechanisms governing macrophages becomes imperative to identify metabolic targets that can potentially impact different stage of diseases prognosis (14–17).

The purpose of this review is to provide a comprehensive overview of the current understanding of the metabolic processes governing macrophages in different states of polarization and activation. Specifically, within the context of NAFLD/NASH, we will examine and analyze the latest findings pertaining to the regulation of macrophage metabolism. Additionally, we will explore potential metabolic targets for therapeutic interventions and strategies to modulate macrophage metabolism in the management of NAFLD/NASH.

## 2 Macrophages in NAFLD and NASH

### 2.1 Macrophage polarization

**Figure 1:**
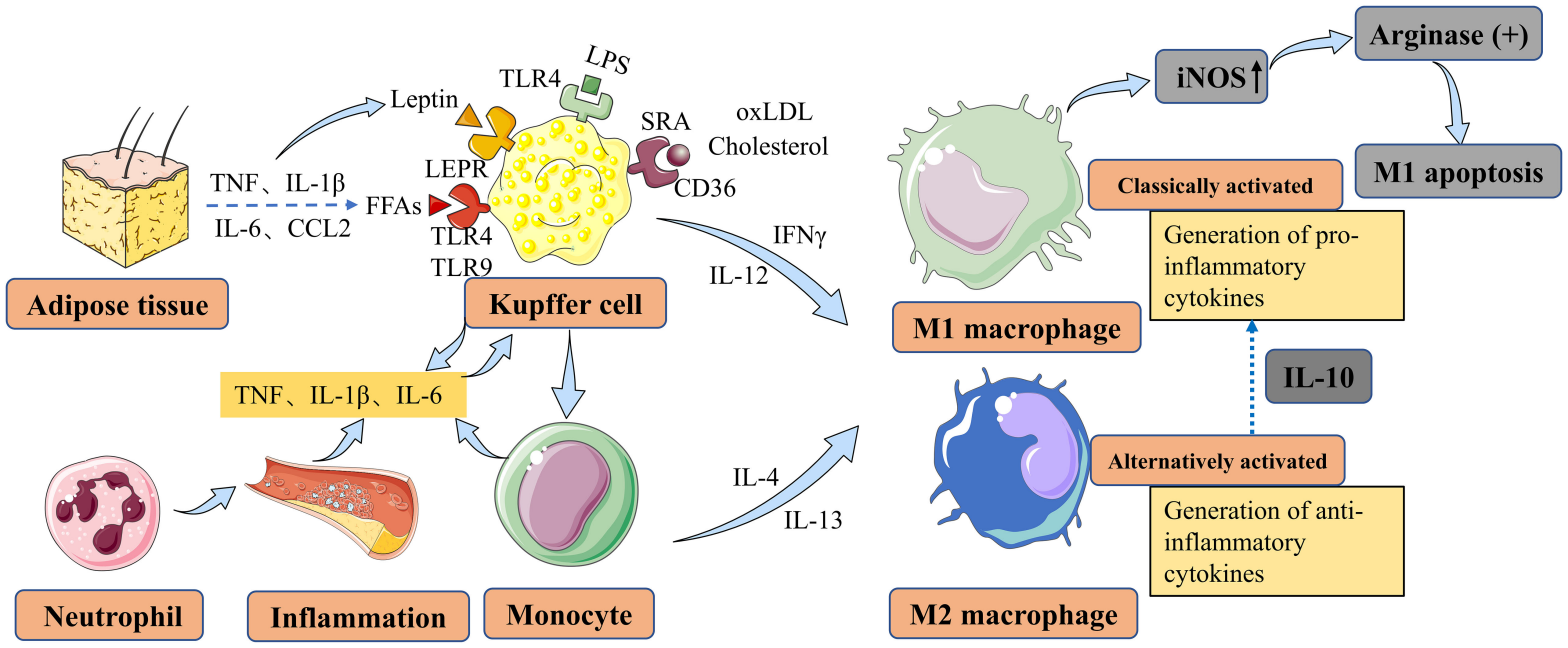
Main factors contributing to macrophage polarization and activation in NAFLD and NASH. Liver macrophages are derived either from resident KCs or from recruited circulating monocytes. *In vitro*, monocytes can be polarized into M1-type or M2-type macrophages, which are associated with classical and alternative activation, respectively. M1 macrophages contribute to inflammation in NASH, while M2 macrophages exert anti-inflammatory effects. M2 macrophages secrete IL10, which selectively induces cell death in M1 KCs expressing high levels of iNOS, and this process involves the activation of arginase. KCs can be activated by LPS through TLRs, FFAs through TLRs, leptin through LEPR originating from adipose tissue, and cholesterol and oxLDL through CD36 and SRA in the context of NAFLD/NASH. KCs secrete TNF, IL-1β, and IL-6 to maintain neutrophil homeostasis. Monocytes differentiate into M1 macrophages, further exacerbating hepatic inflammation in NAFLD. NAFLD, nonalcoholic fatty liver disease; NASH, nonalcoholic steatohepatitis; KCs, Kupffer cells; IL-10, interleukin-10; iNOS, inducible nitric oxide synthase; LPS, lipopolysaccharide; TLRs, toll-like receptors; FFAs, free fatty acids; LEPR, leptin receptor; oxLDL, oxidized low-density lipoprotein; SRA, scavenger receptor A; TNF, tumor necrosis factor; IL-1β, interleukin-1 beta; IL-6, interleukin-6; CCL2, CC-chemokine ligand 2; IFNγ, interferon-gamma; IL-4, interleukin-4; IL-12, interleukin-12; IL-13, interleukin-13.

Macrophage polarization refers to the distinct activation state of macrophages at a specific time and location (18–20). However, it should be noted that macrophage polarization is not a static or fixed state, as macrophages exhibit high plasticity and the ability to integrate diverse signals from damaged tissue, microorganisms, and normal tissue environments. This integration of signals leads to the development of dynamic and unstable polarization states. The regulation of macrophage polarization involves multiple pathways, including epigenetic and cell survival mechanisms that govern macrophage maturation and longevity. Furthermore, the tissue microenvironment and external factors such as microbial products and inflammation-related cytokines play crucial roles in macrophage polarization (**Figure 1**). These pathways collectively determine the specific polarization state assumed by macrophages (21).

**Figure 2:**
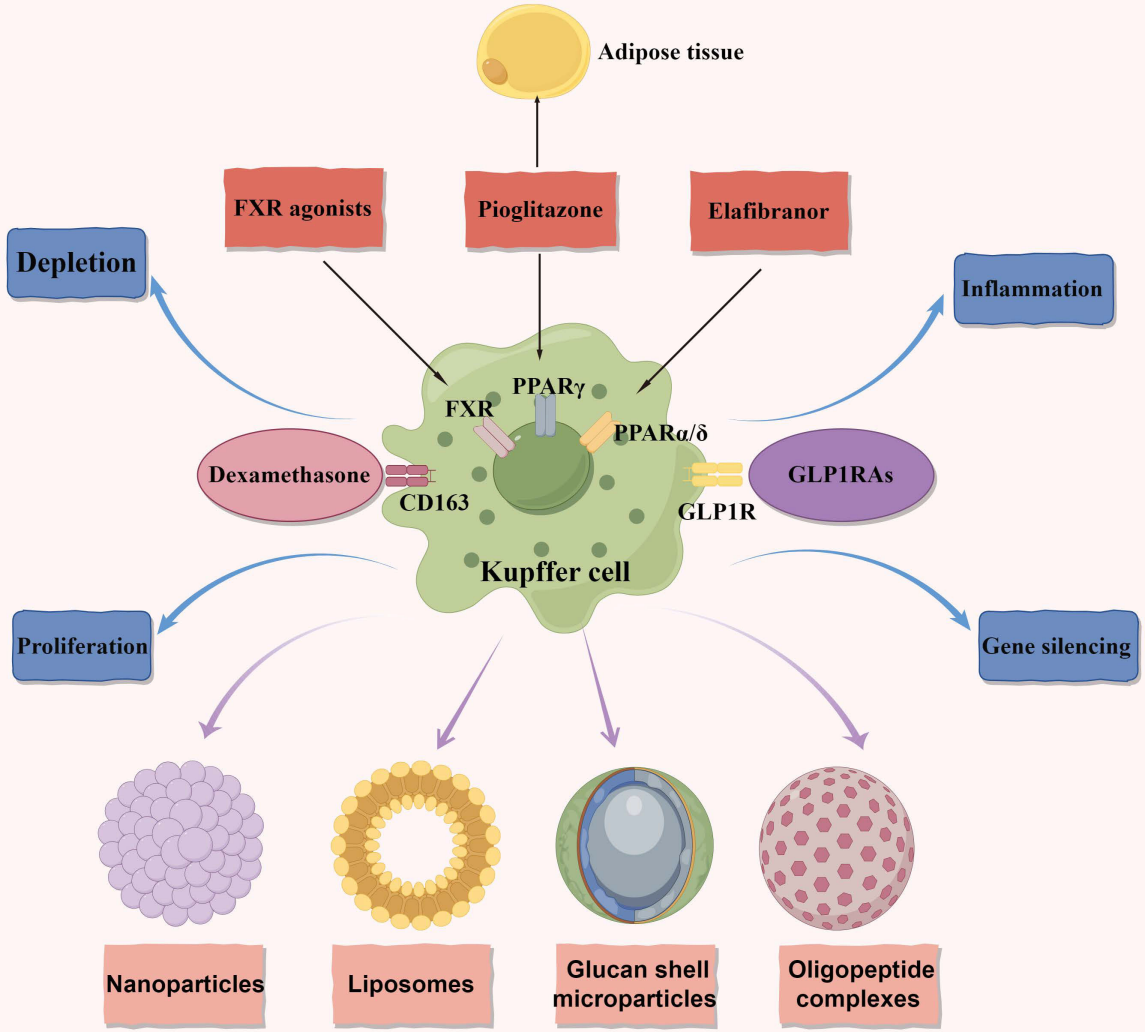
Macrophage metabolism as therapeutic targets in NAFLD and NASH. The figure primarily focuses on therapeutic targets related to macrophage metabolism and illustrates four delivery methods. Additionally, it represents four distinct therapeutic approaches. Anti-CD163-dexamethasone involves delivering the corticosteroid dexamethasone through the CD163 receptor. FXR agonists exert anti-inflammatory and anti-fibrotic effects. In macrophages, they reduce the production of pro-inflammatory cytokines and promote the polarization of macrophages towards an anti-inflammatory phenotype. GLP1RAs have multiple targets acting on the GLP1R. They decrease macrophage infiltration in NAFLD and promote the polarization of macrophages towards an anti-inflammatory phenotype. Pioglitazone acts on the PPARγ and exerts anti-inflammatory and anti-fibrotic effects in NAFLD. It affects both adipose tissue and the liver. Elafibranor is a dual agonist of PPARα and PPARδ. It regulates metabolic homeostasis and inflammation in the liver and adipose tissues, leading to the resolution of NASH. It also reduces macrophage infiltration and promotes an anti-inflammatory macrophage phenotype. NAFLD, nonalcoholic fatty liver disease; NASH, nonalcoholic steatohepatitis; FXR, the farnesoid X receptor; GLP1RAs, glucagon-like peptide-1 receptor agonists; GLP1R, glucagon-like peptide-1 receptor; PPARγ, peroxisome proliferator-activated receptor gamma; PPARδ, peroxisome proliferator-activated receptor delta; PPARα, peroxisome proliferator-activated receptor alpha.

Macrophages possess the ability to differentiate into various phenotypes, commonly categorized as M1 and M2 types, which often exhibit contrasting characteristics (**Figure 1**). The characteristics and regulation of macrophages are complex and interconnected, relying on the dynamic nature of their microenvironment (22). The incomplete characterization of macrophages and their functional polarization in many studies present significant challenges in their interpretation (23, 24). Pharmacological interventions targeting the polarization of macrophages towards an M2 phenotype have shown partial reversal of steatosis and hepatocyte apoptosis (25, 26). However, the efficacy of such interventions may vary depending on the specific microenvironment and the complexity of the underlying pathophysiology. Therefore, further investigation is warranted to gain a deeper understanding of the shared mechanisms governing macrophage polarization and to identify more effective therapeutic approaches for NAFLD/NASH (27). Laboratory studies have demonstrated that M2-type macrophages can induce apoptosis in M1-type macrophages through the activation of the enzyme arginase, mediated by the release of interleukin-10 (IL-10) (28) (**Figure 2**). Animal studies suggest that macrophages exhibiting a pro-inflammatory phenotype contribute to the severity of NAFLD (29, 30). The activation of these macrophages can lead to the production of pro-inflammatory cytokines and the promotion of oxidative stress, ultimately driving the progression of liver fibrosis and other associated complications (31, 32).

The presence of macrophages exhibiting a reparative and anti-inflammatory phenotype in NAFLD has been associated with reduced hepatic injury. These macrophages have the ability to produce cytokines that reduce inflammation and promote tissue healing, thereby aiding in the mitigation of inflammation and improvement of liver function (33–38). An important study has indicated that patients with NASH exhibit higher expression of markers associated with M2 macrophages, suggesting the potential role of these macrophages in the regeneration and repair of liver tissue following hepatocyte damage (39). However, concerns have been raised regarding the potential risk of developing fibrosis as a result of this process (39). Selective targeting of macrophages has shown promise as a therapeutic approach for NASH. Combining the potent corticosteroid dexamethasone with a typical surface marker of M2 macrophages (CD163) has demonstrated enhanced reduction of necroinflammation and fibrosis in a rat model of fructose-induced NASH (**Figure 2**). These positive results suggest that targeting macrophages based on surface markers could be a potential strategy for developing novel treatments for NASH (40). However, further investigation is needed to explore the safety and efficacy of this approach in human subjects. In summary, the examples mentioned above illustrate the diverse spectrum of macrophage polarization observed in NAFLD/NASH, with many of their *in vivo* roles still requiring comprehensive understanding.

### 2.2 Macrophage activation

Macrophages play versatile roles in the human body, including involvement in embryonic development, tissue repair, and inflammation (41). They exhibit remarkable plasticity and can adapt their physical characteristics based on their microenvironment and functional requirements (21, 42). Consequently, macrophages display a spectrum of activation states, characterized by variations in their transcriptome in response to stimuli such as fatty acids, cholesterol, and their metabolites (42, 43). Macrophages encounter a wide range of stimuli in their environment, leading to diverse phenotypes and functions. Traditionally, macrophages have been classified into two main categories: ‘classically activated’ or M1 macrophages and ‘alternatively activated’ or M2 macrophages (44–48), as mentioned earlier. M1 macrophages are responsible for secreting pro-inflammatory cytokines, while M2 macrophages exhibit an anti-inflammatory phenotype (49–51) (**Figure 1**). Macrophages infiltration and Kupffer cells (KCs) activation were found to further express pro-inflammatory cytokines in the NASH model (52). Macrophages dynamically adjust their metabolic characteristics in response to the surrounding microenvironment, enabling them to perform their functions during both homeostasis and inflammation (**Figure 1**). This metabolic adaptation helps maintain a delicate balance between pro-inflammatory and anti-inflammatory responses. The complex process of metabolic reprogramming in macrophages is regulated by factors such as cytokines, growth factors, and nutrient availability, and is crucial for their optimal functioning in both health and disease (53, 54). The focus of this review is specifically on the macrophage response to lipids and their metabolites. Lipid metabolism and the interaction between macrophages and lipid molecules play a significant role in the regulation of macrophage function and their involvement in various diseases and pathological conditions. Understanding the intricacies of how macrophages respond to lipids and their metabolites can provide valuable insights into the development of targeted therapeutic strategies for diseases such as NAFLD/NASH.

#### 2.2.1 FAs

Fatty acids (FAs) can undergo metabolism to produce intermediates that induce liver damage, known as lipotoxicity, which is considered a key mechanism underlying NAFLD progression (55). NAFLD is associated with increased lipolysis in adipose tissues, leading to an elevated influx of free fatty acids (FFAs) into the liver. The increased arrival of FFAs can exacerbate lipotoxicity and contribute to liver damage progression. Therefore, strategies aimed at reducing FAs accumulation or regulating their metabolism could be potential interventions for preventing and treating NAFLD. Experimental studies have shown that different FFAs have distinct effects on macrophages. For instance, in a mouse monocyte-macrophage cell line, it has been demonstrated that saturated fatty acids like lauric and palmitic acids stimulate the toll-like receptor 4 (TLR4) and nuclear factor-κB (NF-κB) pathways, leading to the production of inflammatory mediators such as cyclooxygenase 2 (COX2), inducible nitric oxide synthase (iNOS), and interleukin-1alpha (IL-1α) (56) (**Figure 1**). In contrast, unsaturated fatty acids do not have the same effect (56). Furthermore, it has been shown in animal models that trans-fatty acids diminish the ability of KCs to engulf particles, alongside FFAs. Furthermore, animal models have shown that trans-fatty acids impair the ability of KCs to engulf particles, including FFAs. Additionally, when exposed to lipopolysaccharide (LPS) stimulation, these cells, including KCs, exhibit increased production of tumor necrosis factor (TNF) and macrophage activation in NAFLD (57) (**Figure 1**). In laboratory studies, primary KCs exposed to peroxidized linoleic acid showed elevated levels of the pro-inflammatory mediators iNOS and COX2, along with increased release of TNF (58). Pro-inflammatory cytokines such as TNF, interleukin-1 beta (IL-1β), and IL-6 can be secreted by KCs to regulate neutrophil homeostasis and immune response (59–61) (**Figure 1**). Moreover, in NAFLD, monocytes can differentiate into M1 macrophages, exacerbating hepatic inflammation (34, 62). These findings indicate that oxidized linoleic acid may play a role in the progression of liver inflammation by promoting the activation of KCs, which are crucial immune cells involved in NAFLD development (58, 63) (**Figure 1**). Moreover, KCs can be activated in NAFLD through Toll-like receptors (TLRs) by FFAs and adipokines (**Figure 1**). Importantly, elevated levels of TNF, IL-1β, IL-6, and CC-chemokine ligand 2 (CCL2) have been observed in adipose tissue, further contributing to KC activation through FFAs and leptin in NAFLD (64).

#### 2.2.2 Cholesterol

In addition to FFAs, there is growing recognition of the potential pathological contribution of excess cholesterol to the progression of NAFLD and NASH (65–67). Liver macrophages have significant interactions with cholesterol. Studies using a mouse model of high-fat and high-cholesterol (HFHC) NASH have revealed the formation of hepatic crown-like structures (hCLS) by hepatic macrophages. Lipogranuloma formation occurs when macrophages surround remaining lipid droplets from dead hepatocytes within the hCLS. The activation of macrophages and the release of pro-inflammatory cytokines and other mediators are believed to contribute to the advancement of liver damage in NASH (68, 69) (**Figure 1**). Apart from cholesterol, the activation of KCs can also be mediated by oxidized low-density lipoprotein (oxLDL) through receptors such as CD36 and scavenger receptor A (SRA) (**Figure 1**). Additionally, leptin, one of the prominent adipokines, exerts its effects on KCs through its receptor (LEPR), originating from adipose tissue. This interaction plays a role in inhibiting steatosis and lipogenesis (**Figure 1**). While there is increasing research on macrophage polarization and its role in the development of NAFLD and NASH, most studies have relied on animal models. Consequently, there is a scarcity of human data to fully support these findings. Further investigations using human samples and clinical studies are necessary to validate and expand our understanding of macrophage involvement in NAFLD and NASH in humans.

To summarize, saturated fatty acids, cholesterol, and lipid byproducts have been demonstrated to directly stimulate macrophages and enhance their vulnerability to activation caused by endotoxins, leading to an inflammatory reaction.

### 2.3 KCs and monocyte-derived macrophages

Macrophages, as integral constituents of the innate immune system, display remarkable heterogeneity within the hepatic environment, encompassing liver-resident KCs and recruited monocyte-derived macrophages (70, 71). Both liver-resident KCs and recently recruited monocyte-derived macrophages play pivotal roles in modulating inflammation, fibrogenesis, and fibrolysis in the context of NAFLD and NASH (72). A study has provided evidence supporting a positive correlation between the abundance of KCs in biopsy samples and the severity of NAFLD in patients (73). KCs possess the capacity to attract immune cells that undergo differentiation toward the M1 phenotype, thus eliciting the production of pro-inflammatory cytokines subsequent to liver injury (22, 74–76).

In addition to liver-resident KCs, macrophages derived from monocytes also play a significant role in the progression of NAFLD and NASH. Unlike KCs, recruited monocytes exhibit distinct morphological characteristics, providing evidence for the existence of one of the two major subpopulations of hepatic macrophages in NAFLD (77). The higher presence of CC-chemokine receptor 2^+^ (CCR2^+^) macrophages in patients with more severe NAFLD suggests that monocyte-derived macrophages, rather than KCs, contribute significantly to the pathogenesis of NAFLD (12). This notion is supported by the observation of increased infiltration of monocytes that rapidly differentiate into pro-inflammatory macrophages in an animal model, further highlighting the importance of monocyte-derived macrophages in NAFLD (78). In patients with NASH accompanied by fibrosis and cirrhosis, there was a notable increase in the number of pro-inflammatory macrophages expressing CCR2 in the portal areas, providing further evidence for the involvement of monocyte-derived macrophages in fibrosis development (79).

## 3 Crosstalk between lipid-laden macrophages and KCs

Lipid-laden macrophages, including hepatic stellate cells (HSCs) and KCs, exert their effects in the context of NAFLD and NASH through multifaceted mechanisms. Among the critical cytokines produced, transforming growth factor-beta (TGF-β) plays a significant role in the pathogenesis of inflammation and fibrosis in NAFLD (80). Liver macrophages play a pivotal role in fibrosis development in NAFLD and other liver diseases, as they release cytokines such as IL-6 and TGFβ, which serve as activators of HSCs and myofibroblasts (81–83).

Leptin and adiponectin, prominent adipokines, exhibit interactions not only with liver macrophages but also directly with HSCs. In the context of NAFLD, elevated levels of leptin have been observed, and they are correlated with disease severity. Leptin acts through its receptor and demonstrates potential anti-steatotic properties by enhancing fatty acid oxidation and suppressing hepatic *de novo* lipogenesis (84). However, it is noteworthy that leptin also contributes to the exacerbation of hepatic inflammation and fibrosis in NAFLD (84). On the contrary, adiponectin levels are reduced in individuals with NAFLD, while elevated levels appear to confer protection against obesity, NAFLD, and NASH (85). Through its interaction with adiponectin receptor protein 1 and 2, adiponectin activates AMP-activated protein kinase and induces peroxisome proliferator-activated receptor alpha (PPARα), thereby promoting fatty acid oxidation and reducing hepatic steatosis. Additionally, adiponectin alleviates hepatic inflammation and fibrosis by inhibiting the proliferation and migration of activated HSCs, among other effects (86). Leptin is known to contribute to the fibrogenic phenotype of macrophages. Previous research has demonstrated that leptin can enhance the expression of the primary pro-fibrogenic cytokine TGFβ1 in isolated Kupffer cells, potentially through the involvement of the leptin receptor (87). A more comprehensive investigation conducted revealed that leptin induces the upregulation of TGFβ1 and connective tissue growth factor in KCs, with this effect being dependent on the presence of the leptin receptor and involving the activation of signal transducer and activator of transcription 3 (STAT3) and NF-κB, among other factors (88). This process leads to increased activation of quiescent HSCs, resulting in amplified expression of fibrogenic genes, notably TGFβ1. Notably, leptin has the ability to directly stimulate fibrogenesis by activating HSCs both *in vitro* and *in vivo* (89). These findings indicate that leptin possesses a potent pro-fibrogenic effect, as it induces the expression of pro-inflammatory and pro-fibrotic genes in KCs and directly drives HSC-mediated fibrogenesis.

## 4 Macrophage metabolism as therapeutic targets in NAFLD/NASH

In NASH, a model of liver injury, the liver’s capacity to process excessive amounts of sugars and fats, which are the main metabolic energy sources, becomes impaired. This condition, known as substrate-overload lipotoxicity, leads to the accumulation of harmful lipid species (90–92). The presence of these metabolites can induce stress in liver cells, resulting in damage and eventual cell death. Over time, this process can contribute to the development of fibrosis and genetic instability (25, 93, 94). Consequently, individuals with NASH are at an increased risk of developing cirrhosis and HCC. Macrophages play a significant role in various inflammatory conditions and have a crucial impact on the progression and prognosis of these diseases. In the context of NAFLD, macrophages have been found to be key cells influencing disease advancement. Therefore, targeting macrophages has emerged as a promising therapeutic strategy for various disorders, including NAFLD. Recent discoveries in macrophage metabolism in NAFLD have shed light on potential therapeutic interventions. Strategies aimed at targeting macrophage metabolism could help modulate their functions and alleviate the inflammatory processes associated with NAFLD. These strategies may involve manipulating specific metabolic pathways or targeting key enzymes or receptors involved in macrophage metabolism. By understanding and targeting macrophage metabolism, it may be possible to develop novel therapeutic approaches for NAFLD and related conditions. However, further research is needed to fully elucidate the underlying specific mechanisms for NAFLD and evaluate the efficacy and safety of these strategies in clinical settings.

### 4.1 FXR as a therapeutic target on KCs for NAFLD/NASH

KCs play an important role in NAFLD/NASH progression and a promising target for intervention. Indeed, certain medications have the potential to indirectly influence NAFLD by targeting KCs. The farnesoid X receptor (FXR), also known as the bile acid receptor, has shown promising therapeutic benefits in the treatment of NAFLD (**Figure 2**). Clinical trial data supports the effectiveness of obeticholic acid, an FXR agonist, in inhibiting hepatic glucose and lipid metabolism, as well as exhibiting anti-inflammatory and anti-fibrotic properties in NAFLD (95). FXR agonists have been found to decrease the production of pro-inflammatory cytokines in KCs by attenuating liver inflammation induced by LPS (96). Moreover, in both laboratory settings and living organisms, FXR agonists have been shown to transform macrophages into an anti-inflammatory phenotype (97, 98)(**Figure 2**). These findings suggest that the beneficial effects of FXR activators in NASH may be partially due to their impact on KCs. Currently, a comprehensive phase III clinical trial is underway to evaluate the efficacy of obeticholic acid in patients with NASH and fibrosis (4).

### 4.2 GLP1R and PPAR as promising targets on KCs for NAFLD/NASH

Indeed, the use of glucagon-like peptide-1 receptor agonists (GLP1RAs) has shown promise as a potential therapeutic strategy for NASH. GLP1RAs are agonists of the glucagon-like peptide-1 receptor (99–101)(**Figure 2**). In a clinical study, liraglutide demonstrated partial histological improvement in NASH (102). Furthermore, dipeptidyl peptidase 4 inhibitors, which indirectly activate the glucagon-like peptide-1 (GLP1) receptor, have been found to decrease the number of pro-inflammatory monocytes in the liver and shift macrophage polarization towards the M2 anti-inflammatory phenotype in mice fed a methionine-choline-deficient (MCD) diet (103). These findings suggest that GLP1RAs hold significant promise for the treatment of NASH by influencing inflammatory pathways and improving liver histology (**Figure 2**). Furthermore, the stimulation of peroxisome proliferator-activated receptor gamma (PPARγ) through agonists like pioglitazone promotes the conversion of macrophages into an anti-inflammatory state. This conversion has been shown to alleviate hepatic steatosis by enhancing the uptake and breakdown of fatty acids (104–106). Similarly, peroxisome proliferator-activated receptor delta (PPARδ) plays a crucial role in controlling the polarization of KCs towards the anti-inflammatory M2 phenotype (104) (**Figure 2**). Elafibranor, a dual agonist for PPARα and PPARδ, has shown improved effectiveness in treating NASH compared to a placebo without negatively impacting fibrosis progression (107) (**Figure 2**). These findings collectively suggest that modulating the characteristics of macrophages may represent a viable target for the treatment of NASH. The use of GLP1RAs, PPARγ agonists like pioglitazone, and dual PPARα/PPARδ agonists like elafibranor hold promise in influencing macrophage polarization, inflammatory pathways, and improving liver histology in NASH.

### 4.3 CCR2 and CCR5 as potential treatment strategies on HSCs for NAFLD/NASH

The recruitment of monocytes plays a pivotal role in the advancement of NAFLD and offers a potential avenue for intervention. In an animal model of NASH, the administration of cenicriviroc, a dual antagonist targeting CCR2 and CCR5, exhibited significant improvements in fibrosis and inflammation (108). A subsequent study provided additional evidence of the beneficial effects of cenicriviroc on macrophage numbers and fibrosis in mouse models of NASH (109). In some clinical trials, it was observed that a significantly higher proportion of patients treated with cenicriviroc experienced improvements in fibrosis compared to those in the placebo group. Nevertheless, there was no discernible discrepancy observed among the groups with regards to the primary outcome of attaining a NAFLD activity without exacerbating fibrosis (110). This finding could potentially be ascribed to the presence of both CCR2 and CCR5 on HSCs, as the inhibition of chemokines may have hindered both detrimental and advantageous activation and recruitment of macrophages (111).

### 4.4 Macrophage biomarkers

The proposition that macrophage involvement in the pathogenesis of NAFLD and NASH suggests that markers of macrophage activation could potentially serve as biomarkers for disease severity and treatment response. In a cohort study, the levels of soluble CD36, an indicative marker for macrophage lipid accumulation, were measured (112). The findings revealed elevated levels of soluble CD36 in individuals with impaired glucose regulation, metabolic syndrome, and an increased likelihood of fatty liver, as determined by noninvasive steatosis estimates (113, 114). Moreover, several studies have reported sCD163, a macrophage activation marker specific to certain lineages, as a promising biomarker for predicting liver disease severity (115–119). In two distinct cohorts comprising 195 participants each, sCD163 demonstrated a strong ability to predict advanced fibrosis in adults diagnosed with NAFLD, as evidenced by receiver operating characteristic (AUROC) values of 0.77 and 0.80. Hence, it is reasonable to propose that sCD163 may serve as a distinctive biomarker for macrophages, enabling the anticipation of NASH disease activity, fibrosis, and treatment response. Furthermore, circulating microparticles, originating from activated or apoptotic cells and retaining the surface characteristics of their parent cells, show significant potential as prognostic markers for histological NASH (120). Recent studies have witnessed a notable increase in the investigation of macrophage markers associated with NAFLD/NASH. However, thus far, none of these markers have been examined as predictive biomarkers with clinical outcomes in NAFLD. This observation emphasizes the need for extensive future research in this field.

### 4.5 Therapeutic strategies and delivery pathways

Currently, the majority of therapeutic strategies targeting macrophages rely on receptor-mediated phagocytosis to achieve specificity (121–123). In this approach, compounds are custom-designed to encapsulate therapeutics and possess surface modifications that can be recognized by macrophage receptors (124). Although these receptors are not exclusive to macrophages, they enable the selective identification of cells exhibiting distinct phenotypes and activation states. This receptor-based approach offers a precise and targeted means of delivering therapeutic agents into macrophages, thereby minimizing off-target effects (125). Once inside macrophages, a variety of therapeutic interventions, including depletion, proliferation control, inflammation modulation, and gene silencing, are commonly employed (**Figure 2**). An alternative strategy involves altering the signaling pathways responsible for macrophage-mediated inflammation. Introduction of anti-inflammatory agents into the macrophage cytoplasm allows for the modulation of inflammatory cytokine production and release (126, 127). By employing these therapeutic approaches and utilizing methods that specifically target different subsets of macrophages, a multitude of methodologies can effectively regulate macrophage numbers and improve the state of NASH.

Four primary delivery methods have been identified for targeted administration of therapeutic agents to macrophages: nanoparticles, liposomes, glucan shell microparticles, and oligopeptide complexes (**Figure 2**). The rational approach of utilizing nanoparticles for macrophage-specific drug delivery has been conceptualized and implemented. Although nanoparticle technologies have been developed for targeting macrophage receptors in various diseases, their potential in treating NAFLD/NASH remains unexplored. Similar to nanoparticles, liposome shells can be modified to incorporate ligands or antibodies that selectively target specific macrophage phenotypes based on receptor specificity. Exploiting the inherent phagocytic properties of macrophages (128, 129), liposomes can gain entry into these cells. For instance, clodronate-loaded liposomes have been utilized to induce apoptosis in macrophages upon internalization, leading to their depletion. Insights from fields beyond metabolism in the realm of liposome delivery may offer valuable perspectives for addressing NAFLD/NASH. A recent investigation has explored the use of yeast-derived beta-glucans (Y-BGs) as a distinct encapsulation mechanism with the ability to target macrophages independently of their activation status (130). Administration of Y-BGs orally has been shown to promote the production of IL-10, an anti-inflammatory cytokine (131). Experimental studies have demonstrated that Y-BGs enhance anti-inflammatory activity in macrophages through an IL-10-mediated mechanism (132). Delivering genes to specific tissues without relying on viruses can be challenging, but the application of oligopeptides in conjunction with gene-modulating compounds offers a promising solution for such cases (133–135). This fortuitous finding holds potential for precise transportation of non-viral gene-modifying technology to adipose deposits and adipose tissue macrophages, facilitating targeted outcomes (136).

## 5 Conclusions and future perspectives

Exploring the metabolic activities of macrophages presents new potential for the treatment of NAFLD/NASH. Considering the crucial involvement of macrophages in inflammatory and metabolic disorders, focusing on macrophage metabolism emerges as a promising approach. However, future investigations into macrophage metabolism face certain challenges and considerations. These include accurately targeting macrophages or identifying metabolic targets that do not inadvertently yield positive outcomes. Furthermore, macrophages possess the ability to alter their phenotype and potentially their metabolic state during different stages of disease, which could hinder the effectiveness of metabolic targeting. Nevertheless, a substantial portion of macrophage metabolism research lacks sufficient *in vivo* experimental evidence. Various factors within the microenvironment can influence the metabolism and functionality of macrophages. Thus, employing specialized experimental techniques will be crucial for advancing macrophage metabolism studies into an *in vivo* context. It is anticipated that significant breakthroughs in macrophage metabolism will lead to therapeutic targets capable of influencing disease outcomes in NAFLD/NASH.

## Author contributions

WZ: Conceptualization, Investigation, Software, Supervision, Validation, Visualization, Writing – original draft, Writing – review & editing. RL: Conceptualization, Data curation, Investigation, Resources, Software, Supervision, Validation, Visualization, Writing – review & editing.
